# The combined impact of diet, physical activity, sleep and screen time on academic achievement: a prospective study of elementary school students in Nova Scotia, Canada

**DOI:** 10.1186/s12966-017-0476-0

**Published:** 2017-03-09

**Authors:** Erin L. Faught, John P. Ekwaru, Douglas Gleddie, Kate E. Storey, Mark Asbridge, Paul J. Veugelers

**Affiliations:** 1 Population Health Intervention Research Unit, School of Public Health, University of Alberta, 3-50 University Terrace, 8303-112 street, Edmonton, AB T6G 2T4 Canada; 2 Department of Elementary Education, University of Alberta, 436 Education South Tower, 11210 - 87 ave, Edmonton, AB T6G 2G5 Canada; 3 Department of Community Health and Epidemiology, Department of Emergency Medicine, Dalhousie University, Centre for Clinical Research, 4th Floor, 5790 University Avenue, Halifax, Nova Scotia B3H 1V7 Canada

**Keywords:** Diet, Physical activity, Sedentary behavior, Sleep, Children, School health, Epidemiology, Childhood obesity

## Abstract

**Background:**

Few studies have investigated the independent associations of lifestyle behaviors (diet, physical activity, sleep, and screen time) and body weight status with academic achievement. Even fewer have investigated the combined effect of these behaviors on academic achievement. We hypothesize that the combined effect of these behaviors will have a higher impact on academic achievement than any behavior alone, or that of body weight status.

**Methods:**

In 2011, 4253 grade 5 (10–11 years old) students and their parents were surveyed about the child’s diet, physical activity, screen time and sleep. Students’ heights and weights were measured by research assistants. Academic achievement was measured using provincial standardized exams in mathematics, reading and writing, and was expressed as ‘meeting’ or ‘not meeting’ expectations as per standardized criterion. Exams were written 1 year following the measurement of lifestyle behaviors. Lifestyle behaviors were measured with self- and parental proxy reports and expressed as meeting recommendations (yes/no) for each behavior. Mixed effects logistic regression models adjusting for demographic confounders and caloric intake were used to determine the independent and combined associations.

**Results:**

Meeting dietary recommendations was associated with increased likelihood of meeting academic expectations for each of math, reading and writing. Meeting recommendations for screen time and sleep was associated with meeting expectations for writing. For all three subjects, meeting additional lifestyle behavior recommendations was associated with higher likelihood of meeting expectations. Children who met 7–9 lifestyle behavior recommendations had greater than three-times the odds of meeting expectations for reading compared to those who met 0–3 recommendations (OR: 3.07, 95% CI: 2.09, 4.51), and 1.47 and 2.77 times the odds of meeting expectations in mathematics and writing, respectively. Body weight status was not associated with academic achievement.

**Conclusions:**

We found that lifestyle behaviors, not body weight status, are strongly associated with student academic performance. Promoting compliance with established healthy lifestyle recommendations could improve both the health and educational outcomes of school-aged children. School-based health promotion initiatives that target multiple lifestyle behaviors may have a greater effect on academic achievement than those that focus on a single behavior.

**Electronic supplementary material:**

The online version of this article (doi:10.1186/s12966-017-0476-0) contains supplementary material, which is available to authorized users.

## Background

It is established that academic success in childhood and adolescence is a strong predictor of future wealth, productivity and health [1, 2]. Provided this, attention to children’s academic achievement must be taken into consideration by public health decision makers aiming to prevent chronic diseases and improve health across the lifespan. This includes not only the resources devoted to educational attainment, but an understanding of the indirect lifestyle factors the help to shape childhood and adolescent academic success.

Healthy diets, sufficient physical activity and sleep, and minimal screen time contribute to a healthy lifestyle, are important to children’s cognitive performance during development, and may potentially optimize academic success [3–11]. Findings from studies investigating the relationship between individual lifestyle behaviors and academic achievement have demonstrated that children with healthy lifestyle behaviors perform better academically [10, 12–15]. In particular, reductions in children’s time spent in physical education has motivated substantial study of the relationship between physical activity and academic achievement [16]. However, few studies have investigated the independent association of multiple lifestyle behaviors with academic achievement; particular the potential confounding in the observed relationship given the strong correlation between lifestyle factors [17–20]. For example, levels of sleep are associated with academic achievement, yet screen time has been shown to be associated with both sleep and academic achievement [14, 21, 22]. If screen time is not taken into consideration when evaluating the association between sleep and academic achievement, the observed relationship may, in part, have contributed to the effects of screen time.

Some scholars have hypothesized that healthy lifestyle behaviors do not act in isolation in their relationship with academic achievement, and that the effects of exhibiting multiple healthy lifestyle behaviors may be greater than the sum of their individual effects [17, 23, 24]. To our knowledge, only two studies have investigated this hypothesis, and both have found convincing evidence in support of it [17, 25]. Using self-reported health data in a cohort of Spanish adolescents, Martinez-Gomez et al. (2014) found that meeting recommendations for 3–4 lifestyle behaviors was associated with higher odds achieving passing grades in Language and Literature and Math, in girls only, compared to those that met fewer recommendations [17]. In a sample of American children in a low-income urban distract, Ickovics et al. found that children exhibiting the largest number of healthy lifestyle behaviors, including a healthy body weight, were more than twice as likely to meet goal on standardized exams compared to those with the fewest [25]. Further investigation of this hypothesis is merited to inform effective health promotion in children, particularly school-based initiatives. Many health promotion initiatives based in schools have focused on singular components of a healthy lifestyle; addressing multiple behaviors simultaneously may produce cumulative benefits that impact both health and academic outcomes.

Our aim is to investigate the independent and combined effects of lifestyle behaviors, as well as body weight status, on children’s academic achievement on standardized exams using a large, population-based sample of grade 5 students from Nova Scotia, Canada. We aim to complement and expand on previous work by using a large, population-based sample as well as including a wide range of dietary components, sleep duration and adherence to established recommendations for sleep, objectively measured heights and weights, and standardized exam results in addition to measures of physical activity and screen time. We evaluate children’s lifestyle behaviors by their adherence to established health recommendations from Health Canada, the United States Departments of Health and Human Services and Agriculture, and the World Health Organization (WHO) for each behavior in order to improve interpretability and applicability of results for public health decision makers [26–28]. We hypothesize that each lifestyle behavior of interest (diet, physical activity, screen time and sleep) will have an independent effect on academic achievement. We also hypothesize that the combined effect of multiple healthy lifestyle behaviors will have a greater effect on academic achievement than their respective individual effects.

## Methods

The 2011 Children’s Lifestyle And School performance Study (CLASS) is a population-based survey examining lifestyle behaviors, weight status, and academic achievement of grade 5 (mostly 10–11 years old) students in Nova Scotia, Canada. All grade 5 students in Nova Scotia, their parents or guardians, and school administrators were invited to participate in the study. Of all schools that had grade 5 classes in the province, principals in 269 of 286 (94.1%) schools provided consent for participation. Following consent from the principal, home packages, including consent forms, were sent home to all parents and guardians of grade 5 children in the school. Parental consent to participate in the survey was provided for 6591 students out of 8736 that were distributed, resulting in an average response rate of 75.4% per school. Of these, 1169 (17.7%) were absent the day of the survey, did not complete the survey, or had caloric intakes <500 or >5000 kcal as these values are considered unrealistic [29], and as such were excluded from analysis, leaving 5422 eligible students. Of remaining students, 4253 (78%) could be successfully linked with their achievement on grade 6 standardized exams in Reading, Writing and Mathematics that were written one full year following the lifestyle behavior assessment; the resulting overall completion rate was 64.5%. Further information and the survey used can be found at http://www.nsclass.ca.

### Data collection

Trained research assistants travelled to participating schools to administer surveys during classroom time. Students completed two surveys. The student survey contained questions about habitual physical activity and personal perceptions about diet. Students also completed the Harvard Food Frequency Questionnaire for Youth/Adolescents (YAQ), a 147-item validated questionnaire adapted for Canadian use that measures habitual intake over the past 12 months [30]. Research assistants measured students’ heights to the nearest 0.1 cm and weights to the nearest 0.1 g using calibrated stadiometers and scales as per standard protocol [31]. Parents completed a home survey reporting on children’s sleep habits and screen time usage, as well questions on household income and parental level of education.

### Exposures

#### Diet

We employed the Harvard Food Frequency Questionnaire YAQ to evaluate students dietary consumption. The YAQ contains 147 questions, 135 of which are regarding specific food items and 11 of which relate to food habits (eating in front of the TV, etc.), about the frequency of consuming items over the past year [30]. Nutrient information was derived using the Canadian Nutrient File (CNF) [32], a Canadian nutrient composition database for commonly consumed foods in Canada. Students’ consumption was evaluated with respect to meeting age-specific recommendations from Health Canada’s *Eating Well with Canada’s Food Guide* [26]. This includes recommendations for daily servings of vegetables and fruit (6 servings), grain products (6 servings), milk and alternatives (3–4 servings) and meat and alternatives (2 servings). The Canadian Food Guide does not have specific recommendations for saturated fat and free sugar intake. Instead we used recommendations developed for American youth for saturated fat intake (<10% of total energy intake) [28], and for free sugars consumption – representing sugars that are added during food processing, not naturally occurring sugars such as lactose – we followed 2015 World Health Organization recommendations (<10% of total energy intake) [27]. We calculated energy intake using responses from the YAQ.

#### Physical activity

The student survey contained the Physical Activity Questionnaire for Children (PAQ-C) instrument, a self-administered, 10-item physical activity recall instrument [33]. The questionnaire has been validated to measure general levels of moderate-to-vigorous physical activity in children aged 8–14 [33–35]. A score between 0 and 5 was calculated from responses for each student, with higher scores indicating higher levels of physical activity. Cutoff values indicating ‘healthy fit’ or ‘at-risk’ in regards to cardio-respiratory fitness for the PAQ-C have been established for children: 2.7 for girls and 2.9 for boys [36]. As such, meeting recommendations for physical activity that correspond with healthy cardio-respiratory fitness were assessed using these cutoff values.

#### Screen time

Parents were asked: On average, about how many hours per day does your Grade 5 child spend watching TV not including school hours? Possible responses were: Less than 1 h a day, 1–2 h per day, 3–4 h per day, or 5 or more hours per day. Children were described as meeting sedentary behavior recommendations if total screen time from television watching was less than 2 h per day as per the Canadian Sedentary Behavior Guidelines [37].

#### Sleep

Parents reported habitual wake up and bed times for children on usual weekdays and weekends. Parents were asked: At what time does your child usually wake up during a) the week (Monday to Friday) and b) the weekend (Saturday and Sunday)? Possible responses were before 6:30 am, 6:30–7:00 am, 7:00–7:30 am, 7:30–8:00 am, 8:00–8:30 am, 8:30–9:00 am and after 9:00 am. Parents were also asked: At what time does your child usually go to bed during a) the week (Sunday to Thursday) and b) the weekend (Friday and Saturday)? Possible responses were before 8:00 pm, 8:00–8:30 pm, 8:30–9:00 pm, 9:00–9:30 pm, 9:30–10:00 pm, 10:00–10:30 pm, and after 10:30 pm. Finally, sleep duration was calculated based on usual bed and wake up times with usual time to fall asleep subtracted for each of weekday and weekend days. Average nightly sleep duration for a typical week was calculated from the mean sleep duration of five weekdays and two weekend days. Students were described as meeting sleep duration recommendations if average duration was between 9 and 11 h, as recommended by the National Sleep Foundation [38].

#### Body weight status

Children’s body mass index (BMI) was calculated using measured heights and weights. Body weight status was assessed using the International Obesity Task Force (IOTF) age- and gender-specific BMI cutoffs for overweight and obesity [39].

#### Potential confounders

Analyses were adjusted for child’s gender, parental education, and household income as assessed by categorical questions in the parental home survey as well as region of residence (urban or rural) determined by postal code. Energy intake was included in analyses that included YAQ data as is recommended [29].

### Outcome

#### Academic achievement

The Nova Scotia Department of Early Education and Childhood Development provided results for standardized provincial exams written by participants in grade 6 (spring 2012), 1 year following the measurement of other variables (spring 2011), in the subjects of Mathematics, Reading, and Writing. Results were provided as dichotomous values of ‘meeting expectations’ and ‘not meeting expectations’. The Nova Scotia Department of Early Education and Childhood Development, who administers the exams, provides standardized rubrics for the exams to determine of children are meeting and not meeting expectations. Teachers from across the province are invited to assist in the marking of assessments. Further information about this process can be found at https://plans.ednet.ns.ca/about-plans.

### Analysis

All analyses were weighted for non-response to represent provincial estimates of the grade 5 student population of Nova Scotia. Response weights were calculated based on postal-code level estimates of household data, available from Canadian census data for both participants and non-participants [40]. As response rates were lower among the lowest income deciles, weights were applied to overcome non-response bias from lower-income neighbourhoods in Nova Scotia [40]. We applied mixed effects models due to the clustering of students within schools. Correlations between exposures of interest were evaluated and can be found in the supplementary material (Additional file 1). Univariable logistic regression was first used to assess the associations between each individual student’s lifestyle behaviors, dichotomized into meeting and not meeting recommendations for each, and their academic achievement. Next, we used multivariable models (Model 1) to adjust for potential confounders and body weight status. Models for individual exposures of interest were run including potential confounders (nine separate models for each recommendation). Another model was run with confounders only, producing the results for Model 1 in Tables 2, 3, and 4 for confounders. Finally, we considered all lifestyle behaviors simultaneously in a full model (model 2) to assess independent associations between meeting each lifestyle behavior recommendation, body weight status, and academic achievement.

To assess combined effects of meeting each lifestyle behavior recommendation, we also considered the effect of the number of recommendations met up to 9 (vegetables and fruit, grain products, milk and alternatives, meat and alternatives, saturated fat, free sugars, physical activity, sleep, and screen time). As with assessing independent associations, univariable and multivariable regression models were employed treating the score as both categorical and continuous to assess the cumulative impact of meeting lifestyle behavior recommendations and academic achievement. This analysis was conducted treating the score as a continuous variable and by splitting scores into three categories, low (meeting 1–3 recommendations), medium (meeting 4–6 recommendations) and high (meeting 7–9 recommendations). All analyses were conducted using Stata version 14.1 IC (StataCorp, Texas, USA).

## Results

Table 1 shows that 87.4% of students met expectations for reading, 89.1% met expectations for writing, and 70.6% met expectations for mathematics. The percentage of children meeting selected lifestyle behavior recommendations was: 32.2% for Vegetables and Fruits, 20.8% for Grain Products, 56.0 for Milk and Alternatives, 86.4 for Meat and Alternatives, 54.3% for saturated fat intake, 62.6% for free sugars intake, 76.7% for physical activity as per the PAQ-C, 91.1% for sleep duration, and 77.8% for screen time. Meeting recommendations for milk and alternatives, meat and alternatives, free sugars, sleep, and screen time all had significant univariate associations with meeting expectations for mathematics (Table 2, Model 1), while vegetables and fruit, grain products, milk and alternatives, meat and alternatives, saturated fat, and free sugars all had significant univariate associations with meeting expectations for reading (Table 3, Model 1). Meeting recommendations for vegetables and fruit, meat and alternatives, free sugars, physical activity, sleep and screen time all had significant univariate associations with meeting expectations for writing. Parental level of education, household income, and gender were all significantly associated with the likelihood of meeting expectations for each subject, while obesity only had a significant univariate association with meeting expectations in mathematics.

**Table 1:** Sample characteristics of grade 5 students participating in the CLASS II Project in Nova Scotia, Canada

Characteristics	*n* = 4253
Age, mean (SD), years	11.0 (0.4)
Proportion Male (sex), %	47.2
Overweight or obese, %	34.7
Energy Intake, mean (SD), kcal	1849.9 (808.1)
Percentage of children who are meeting lifestyle behavior recommendations, %	%
Vegetables and Fruits (6 servings)	32.2
Grain Products (6 servings)	20.8
Milk and Alternatives (3–4 servings)	56.0
Meat and Alternatives (1–2 servings)	86.4
Saturated Fat Intake (<10% total energy)	54.3
Free Sugars (<10% total energy)	62.6
Physical Activity (PAQ-C Score >=2.7 for girls, >=2.9 for boys)	76.7
Sleep Duration (9–11 h)	91.1
Screen Time (<=2 h day of television)	77.8
Percentage of children meeting multiple lifestyle behavior recommendations, %	%
Low (1–3)	7.9
Medium (4–6)	64.7
High (7–9)	27.5
Academic Achievement, % meeting expectations	%
Reading	87.4
Writing	89.1
Mathematics	70.6
Parental Education, %	%
Secondary or Less	17.3
College Diploma	38.4
University or Graduate degree	36.3
Missing/Prefer not to answer	8.0
Household Income (CAN$), %	%
<=20,000	20.3
20,001–40,000	13.3
40,001–60,000	24.1
>=60,001	19.8
Missing/Prefer not to answer	22.5
Region of Residence, %	%
Urban	69.7
Rural	30.3

**Table 2:** The associations between adherence to lifestyle behavior recommendations and meeting expectations on a standardized exam in Mathematics

	Academic Achievement in Mathematics					
	Univariable		Model 1*		Model 2**	
	OR (95% CI)	*p*-value	OR (95% CI)	*p*-value	OR (95% CI)	*p*-value
Vegetables and Fruits						
No (reference)	-	-	-	-	-	-
Yes	1.13 (0.95, 1.35)	0.18	1.06 (0.90, 1.27)	0.48	1.04 (0.86, 1.27)	0.66
Grain Products						
No (reference)	-	-	-	-	-	-
Yes	1.02 (0.86, 1.22)	0.81	1.00 (0.83, 1.20)	0.99	0.99 (0.82, 1.20)	0.92
Milk and Alternatives						
No (reference)	-	-	-	-	-	-
Yes	**1.34 (1.15, 1.57)**	**<0.001**	**1.27 (1.09, 1.49)**	**0.002**	**1.33 (1.11, 1.59)**	**0.002**
Meat and Alternatives						
No (reference)	-	-	-	-	-	-
Yes	**1.56 (1.27, 1.90)**	**<0.001**	**1.56 (1.28, 1.91)**	**<0.001**	**1.59 (1.27, 1.99)**	**<0.001**
Saturated Fat						
No (reference)	-	-	-	-	-	-
Yes	0.97 (0.82, 1.14)	0.71	0.94 (0.79, 1.12)	0.49	1.00 (0.83, 1.21)	0.99
Free Sugars <10% total kcal						
No (reference)	-	-	-	-	-	-
Yes	**1.63 (1.38, 1.93)**	**<0.001**	**1.33 (1.12, 1.57)**	**0.001**	1.20 (0.99, 1.45)	0.06
PAQ-C						
No (reference)	-	-	-	-	-	-
Yes	1.14 (0.96, 1.35)	0.12	1.10 (0.92, 1.30)	0.30	1.12 (0.94, 1.33)	0.21
Sleep						
No (reference)	-	-	-	-	-	-
Yes	**1.44 (1.01, 2.02)**	**0.04**	1.26 (0.88, 1.80)	0.20	1.42 (0.97, 2.07)	0.71
Screen (TV)						
No (reference)	-	-	-	-	-	-
Yes	**1.33 (1.11, 1.60)**	**0.002**	1.11 (0.92, 1.35)	0.28	1.02 (0.83, 1.25)	0.86
Body Weight Status						
Normal Weight (reference)	-	-	-	-	-	-
Overweight or Obese	0.95 (0.79, 1.13)	0.533	1.00 (0.84, 1.20)	0.97	0.98 (0.82, 1.17)	0.81
Gender						
Girl (reference)	-	-	-	-	-	-
Boy	1.09 (0.93, 1.28)	0.27	1.08 (0.92, 1.27)	0.22	1.11 (0.94, 1.30)	0.22
Parental Education						
Secondary or Less (ref)	-	-	-	**-**	-	-
College Diploma	**1.50 (1.20, 1.86)**	**<0.001**	**1.32 (1.07, 1.63)**	**0.01**	**1.32 (1.06, 1.65)**	**0.01**
University or Graduate Degree	**3.28 (2.65, 4.06)**	**<0.001**	**2.38 (1.91, 2.97)**	**<0.001**	**2.45 (1.96, 3.07)**	**<0.001**
Household Income (CAN$)						
<=20,000 (reference)	-	-	-	-	-	-
20,001–40,000	**1.61 (1.30, 2.01)**	**<0.001**	**1.43 (1.15, 1.79)**	**0.002**	**1.42 (1.13, 1.79)**	**0.003**
40,001–60,000	**2.03 (1.63, 2.53)**	**<0.001**	**1.49 (1.21, 1.85)**	**<0.001**	**1.44 (1.15, 1.80)**	**0.001**
>=60,001	**3.43 (2.71, 4.35)**	**<0.001**	**2.05 (1.58, 2.65)**	**<0.001**	**2.03 (1.56, 2.63)**	**<0.001**
Region of Residence						
Rural (reference)	-	-	-	-	-	-
Urban	0.82 (0.66, 1.04)	0.10	**0.70 (0.59, 0.84)**	**<0.001**	**0.72 (0.58, 0.89)**	**0.002**

*Model 1 adjusted for gender, parental education, household income, and region of residence. Results for Model 1 for confounders are the result of a model using only confounders as predictors

**Model 2 adjusted for above, weight status and all lifestyle behaviors

Results in bold are statistically significant (*p* < 0.05)

**Table 3:** The associations between adherence to lifestyle behavior recommendations and meeting expectations on a standardized exam in Reading

	Academic Achievement in Reading					
	Univariable		Model 1*		Model 2**	
	OR (95% CI)	*p*-value	OR (95% CI)	*p*-value	OR (95% CI)	*p*-value
Meets Vegetables and Fruits						
No (reference)	-	-	-	-	-	-
Yes	**1.44 (1.13, 1.83)**	**0.003**	**1.30 (1.02, 1.66)**	**0.03**	1.19 (0.91, 1.56)	0.20
Meets Grain Products						
No (reference)	-	-	-	-	-	-
Yes	**1.49 (1.13, 1.97)**	**0.005**	**1.49 (1.12, 1.98)**	**0.006**	**1.57 (1.16, 2.14)**	**0.004**
Meets Milk and Alternatives						
No (reference)	-	-	-	-	-	-
Yes	**1.25 (1.02, 1.53)**	**0.03**	**1.24 (1.00, 1.53)**	**0.045**	**1.46 (1.16, 1.84)**	**0.001**
Meets Meat and Alternatives						
No (reference)	-	-	-	-	-	-
Yes	**1.55 (1.20, 2.01)**	**0.001**	**1.55 (1.19, 2.01)**	**0.001**	**1.56 (1.18, 2.08)**	**0.04**
Meets Saturated Fat						
No (reference)	-	-	-	-	-	-
Yes	**1.36 (1.13, 1.65)**	**0.001**	**1.28 (1.06, 1.56)**	**0.01**	**1.28 (1.01, 1.62)**	**0.04**
Meets Free Sugars <10% total kcal						
No (reference)	-	-	-	-	-	-
Yes	**1.91 (1.57, 2.32)**	**<0.001**	**1.56 (1.28, 1.90)**	**<0.001**	**1.29 (1.02, 1.62)**	**0.03**
Meets PAQ-C						
No (reference)	-	-	-	-	-	-
Yes	1.14 (0.90, 1.45)	0.28	1.05 (0.82, 1.34)	0.70	1.05 (0.82, 1.36)	0.66
Meets Sleep						
No (reference)	-	-	-	-	-	-
Yes	1.33 (0.89, 2.00)	0.16	1.21 (0.79, 1.88)	0.38	1.39 (0.90, 2.13)	0.14
Meets Screen Time						
No (reference)	-	-	-	-	-	-
Yes	1.17 (0.94, 1.46)	0.16	1.03 (0.82, 1.31)	0.77	0.96 (0.75, 1.23)	0.74
Body Weight Status						
Normal Weight (reference)	-	-	-	-	-	-
Overweight or Obese	1.01 (0.81, 1.26)	0.92	1.09 (0.86, 1.37)	0.48	1.03 (0.82, 1.31)	0.79
Gender						
Girl (reference)	-	-	-	-	-	-
Boy	**0.56 (0.46, 0.67)**	**<0.001**	**0.57 (0.46, 0.69)**	**<0.001**	**0.57 (0.47, 0.70)**	**<0.001**
Parental Education						
Secondary or Less (ref)	-	-	-	-	-	-
College Diploma	**1.42 (1.09, 1.84)**	**0.008**	**1.33 (1.02, 1.73)**	**0.04**	1.24 (0.93, 1.66)	0.14
University or Graduate Degree	**2.56 (1.92, 3.41)**	**<0.001**	**1.89 (1.40, 2.55)**	**<0.001**	**1.89 (1.37, 2.60)**	**<0.001**
Household Income (CAN$)						
<=20,000 (reference)	-	-	-	-	-	-
20,001–40,000	1.31 (0.97, 1.77)	0.78	1.20 (0.90, 1.62)	0.22	1.23 (0.89, 1.70)	0.21
40,001–60,000	**1.68 (1.20, 2.34)**	**0.002**	1.32 (0.95, 1.84)	0.10	1.33 (0.94, 1.88)	0.11
>=60,001	**3.10 (2.13, 4.51)**	**<0.001**	**1.95 (1.33, 2.87)**	**0.001**	**2.06 (1.37, 3.08)**	**<0.001**
Region of Residence						
Rural (reference)	-	-	-	-	-	-
Urban	1.23 (0.96, 1.57)	0.11	1.03 (0.84, 1.25)	0.81	1.11 (0.87, 1.41)	0.41

*Model 1 adjusted for gender, parental education, household income, and region of residence. Results for Model 1 for confounders are the result of a model using only confounders as predictors

**Model 2 adjusted for above, weight status and all lifestyle behaviors

Results in bold are statistically significant (*p* < 0.05)

After adjusting for potential confounders, meeting recommendations for milk and alternatives, meat and alternatives, and free sugars continued to have significant positive associations with meeting recommendations for mathematics (Table 2, Model 1). Where meeting expectations for reading was the outcome, meeting recommendations for vegetables and fruit, grain products, milk and alternatives, meat and alternatives, saturated fat and free sugars were all associated with increased likelihood of meeting expectations (Table 3, Model 1). Finally, meeting recommendations for meat and alternatives, free sugars, sleep, and screen time were all associated with increased likelihood of meeting expectations for writing (Table 4, Model 1). Body weight status did not have an association with any outcome in these models.

**Table 4:** The associations between adherence to lifestyle behavior recommendations and meeting expectations on a standardized exam in Writing

	Academic Achievement in Writing					
	Univariable		Model 1*		Model 2**	
	OR (95% CI)	*p*-value	OR (95% CI)	*p*-value	OR (95% CI)	*p*-value
Meets Vegetables and Fruits						
No (reference)	-	-	-	-	-	-
Yes	**1.35 (1.06, 1.71)**	**0.02**	1.20 (0.93, 1.54)	0.16	1.08 (0.81, 1.43)	0.61
Meets Grain Products						
No (reference)	-	-	-	-	-	-
Yes	1.05 (0.79, 1.40)	0.72	1.09 (0.82, 1.44)	0.57	1.00 (0.74, 1.37)	0.96
Meets Milk and Alternatives						
No (reference)	-	-	-	-	-	-
Yes	1.15 (0.91, 1.45)	0.25	1.08 (0.85, 1.37)	0.54	1.10 (0.85, 1.43)	0.46
Meets Meat and Alternatives						
No (reference)	-	-	-	-	-	-
Yes	**1.46 (1.08, 1.99)**	**0.01**	**1.40 (1.04, 1.90)**	**0.03**	**1.39 (1.01, 1.91)**	**0.04**
Meets Saturated Fat						
No (reference)	-	-	-	-	-	-
Yes	1.23 (0.99, 1.52)	0.06	1.18 (0.95, 1.48)	0.141	1.16 (0.90, 1.49)	0.25
Meets Free Sugars <10% total kcal						
No (reference)	-	-	-	-	-	-
Yes	**1.66 (1.35, 2.04)**	**<0.001**	**1.43 (1.16, 1.77)**	**0.001**	**1.41 (1.13, 1.49)**	**0.002**
Meets PAQ-C						
No (reference)	-		-	-	-	-
Yes	**1.32 (1.05, 1.66)**	**0.02**	1.19 (0.94, 1.49)	0.14	1.12 (0.88, 1.43)	0.36
Meets Sleep						
No (reference)	-	-	-	-	-	-
Yes	**1.50 (1.05, 2.14)**	**0.03**	**1.56 (1.05, 2.29)**	**0.03**	**1.56 (1.03, 2.34)**	**0.03**
Meets Screen Time						
No (reference)	-	-	-	-	-	-
Yes	**1.47 (1.16, 1.88)**	**0.002**	**1.43 (1.12, 1.83)**	**0.004**	**1.35 (1.04, 1.75)**	**0.03**
Body Weight Status						
Normal Weight (reference)	-	-	-	-	-	-
Overweight or Obese	0.80 (0.63, 1.03)	0.08	0. 84 (0.65, 1.08)	0.17	0.82 (0.64, 1.06)	0.14
Gender						
Girl (reference)	-	-	-	-	-	-
Boy	**0.44 (0.36, 0.55)**	**<0.001**	**0.42 (0.34, 0.52)**	**<0.001**	**0.45 (0.36, 0.56)**	**<0.001**
Parental Education						
Secondary or Less (ref)	-	-	-	-	-	-
College Diploma	1.20 (0.89, 1.63)	0.24	**1.33 (1.07, 1.65)**	**0.01**	1.16 (0.84, 1.61)	0.36
University or Graduate Degree	**1.82 (1.34, 2.46)**	**<0.001**	**2.42 (1.94, 3.03)**	**<0.001**	1.37 (0.96, 1.94)	0.08
Household Income (CAN$)						
<=20,000 (reference)	-	-	-	-	-	-
20,001–40,000	**1.50 (1.07, 2.11)**	**0.02**	**1.46 (1.17, 1.83)**	**0.01**	**1.44 (1.01, 2.06)**	**0.045**
40,001–60,000	**1.65 (1.18, 2.30)**	**0.003**	**1.53 (1.23, 1.90)**	**<0.001**	**1.51 (1.03, 2.21)**	**0.04**
>=60,001	**2.66 (1.80, 3.93)**	**<0.001**	**2.42 (1.94, 3.03)**	**<0.001**	**2.10 (1.31, 3.36)**	**0.002**
Region of Residence						
Rural (reference)	-	-	-	-	-	-
Urban	0.97 (0.74, 1.26)	0.80	**0.70 (0.57, 0.85)**	**<0.001**	0.86 (0.65. 1.12)	0.264

*Model 1 adjusted for gender, parental education, household income, and region of residence. Results for Model 1 for confounders are the result of a model using only confounders as predictors

**Model 2 adjusted for above, weight status and all lifestyle behaviors

Results in bold are statistically significant (*p* < 0.05)

When considering all lifestyle behaviors simultaneously, only meeting recommendations for milk and alternatives, and meat and alternatives remained significantly associated with meeting expectations for mathematics; meeting recommendations for free sugars was borderline significant (Table 2, Model 2: OR: 1.20 [95% CI: 0.99, 1.45]). Meeting expectations for grain products, milk and alternatives, meat and alternatives, saturated fat and free sugars were all positively associated with meeting expectations for reading (Table 3, Model 2). Meeting recommendations for meat and alternatives, free sugars, sleep, and screen time had significant, positive associations with meeting expectations for writing (Table 4, Model 2). Neither overweight nor obesity demonstrated any association with meeting expectations for any subject.

Considering the combined effect of the nine criteria, for each additional criterion met, the odds of meeting the expectations for mathematics was 1.13 times higher (OR: 1.13 [95% CI: 1.06, 1.20]). Table 5 shows that each additional criterion met also increased odds of meeting recommendations for reading by 1.26 times, (OR: 1.26 [95% CI: 1.17, 1.35]), and by 1.21 times for meeting recommendations in writing (OR: 1.21 [95% CI: 1.11, 1.32]).

**Table 5:** Mixed effects logistic regression models of the relationship between children’s combined adherence to multiple lifestyle behavior recommendations and the odds of meeting expectations on standardized exams in Mathematics, Reading and Writing

	Academic Achievement					
	Mathematics		Reading		Writing	
	OR (95% CI) ^a^	*p*-value	OR (95% CI) ^a^	*p*-value	OR (95% CI) ^a^	*p*-value
Per recommendation met^b^	**1.13 (1.06, 1.20)**	**<0.001**	**1.26 (1.17, 1.35)**	**<0.001**	**1.21 (1.11, 1.32)**	**<0.001**
No. of Recommendations Met^b^						
3 or less	-	-	-	-	-	-
4–6	1.12 (0.81, 1.54)	0.50	**1.85 (1.32, 2.59)**	**<0.001**	**1.98 (1.38, 2.84)**	**<0.001**
7–9	**1.47 (1.04, 2.06)**	**0.03**	**3.07 (2.09, 4.51)**	**<0.001**	**2.77 (1.83, 4.20)**	**<0.001**

^a^Adjusted for body weight status, gender, parental education, parental income, region of residence and energy intake

^b^Includes meeting recommendations for vegetables and fruit, grain products, milk and alternatives, meat and alternatives, saturated fat, free sugars, physical activity cutoffs, sleep, and screen time

Results in bold are statistically significant (*p* < 0.05)

The criteria were also considered in groups representing low, medium, and high compliance. Respectively, 7.9, 64.6, and 27.4% were in the low, medium and high categories. Compared to the lowest category, children who were in the highest category had 1.47 times the odds of meeting expectations for mathematics (Table 5: OR: 1.47 [95% CI: 1.04, 2.06]), and 3.07 and 2.77 times the odds for meeting expectations in reading and writing respectively (Table 5: OR: 3.07, [95% CI: 2.09, 4.51], OR: 2.77 [95% CI: 1.83, 4.20]).

## Discussion

We observed that meeting recommendations for diet, sleep and screen time had independent, positive effects for children’s academic achievement. No association was found between meeting physical activity cutoffs and academic achievement. The findings from this study also indicated that the combined effects of meeting multiple lifestyle behavior recommendations had a stronger impact on academic achievement than the individual effects of lifestyle behaviors, particularly for reading and writing. We chose to evaluate lifestyle behaviors based on established recommendations that are widely accepted. Substantial efforts and resources go into the development and promotion of these recommendations, and findings from this study reveal that more efforts are needed to achieve compliance not only for the benefit of health, but for education.

The associations between dietary behaviors and academic achievement are supported by previous literature [6, 7, 41–47]. However, the majority of studies linking diet and academic achievement have tended to focus on breakfast consumption and whole diet, and few studies have evaluated the relationship between established dietary recommendations and academic achievement [7]. In particular, no study has evaluated the relationship between meeting newly released guidelines for free sugar recommendations, which exhibited a strong positive association with each of the three subjects. The lack of association between vegetables and fruit and academic achievement in this study seems inconsistent with much of the literature investigating the association between diet and academic achievement [20, 48]. In previous studies, servings of vegetables and fruit have been assessed as a continuous variable, with more servings being positively associated with academic achievement. In this study, very few children met the recommendation for vegetables and fruit. As such, there may not have been sufficient power to detect any positive effect of meeting recommendations for vegetables and fruit. We conducted additional analyses treating servings of vegetables and fruit as a continuous variable, however, and no significant effect on academic achievement was observed. Milk and alternatives, meat and alternatives, and sugars consumption were more consistent predictors of higher academic achievement. This may be a reflection of a higher income household that is more likely to access and purchase products within these groups more regularly [49].

The observation that meeting designated cutoffs for physical activity levels associated with adequate cardio-respiratory fitness is not significantly associated with higher academic achievement complements existing findings in the literature investigating the relationship of physical activity with children’s school performance. Much of the literature has concluded that the inclusion of more physical activity and physical education relative to other subjects in a school day does not negatively affect school performance [11, 13, 16, 50]. Many studies have found a relationship between children’s physical activity levels and their academic achievement or cognitive development [11, 13, 51–53] though few studies have aimed to investigate its importance independent of other lifestyle behaviors. Another study found that the relationship between physical activity and academic achievement to be curvilinear, suggesting that children who are athletes may have many extra-curricular activities that displace time spent on academics [54]. Though cutoff values are useful in identifying sufficient levels of physical activity for physical health benefits, they may not be the most appropriate way to assess the association between physical activity and academic achievement as this relationship appears to be more complex. An analysis was conducted using PAQ-C score as a continuous variable (results not shown) and physical activity continued to have no effect on academic achievement. However, though the PAQ-C is a well-used and validated questionnaire, its intent is to provide a broad overview of children’s moderate-to-vigorous physical activity levels and it does not provide detailed information about other intensities or frequency of physical activities or the physiological benefits children gain from regular physical activity that would contribute to their academic achievement, among other contributors. In addition, there was a time lag (1 year) between physical activity assessment and exam writing which may have introduced error. The PAQ-C measures regularly moderate-to-vigorous physical activity over a one week period which would not adequately capture the variability in the frequency of physical activity over a 1 year period. As such, this lack of association may be due to the limitations of the measure used, and further investigation using a more detailed means of measurement of children’s physical activity is needed.

Sleep and screen time have been previously associated with academic achievement [10, 14, 55]. This is the second study, to our knowledge, to demonstrate the independent importance of meeting sleep recommendations for these behaviors for academic achievement [17]. In this study, sleep was found to only be associated with performance on writing exams. Sleep has been shown to be crucial for creativity and insight, key determinants of strong writing skills [56]. This study is also among the first to evaluate sleep duration and academic achievement using the National Sleep Foundation guideline [38]. Sleep is recommended within a range of hours, not as a minimum number of hours, and the finding that meeting these sleep recommendations is strongly associated with increased likelihood of meeting academic expectations in writing highlights the importance of both meeting the minimum requirements of sleep while not exceeding the maximum recommended number of hours [38, 57].

Few studies have investigated the association of body weight status and lifestyle behaviors simultaneously to determine their independent associations. Those that have, provide indication that it is not weight status that drives academic achievement, but that both academic achievement and body weight status are a result of long-term lifestyle behaviors [17, 18]. This study complements this important work and indicates that promoting healthy lifestyles and values, rather than focusing on obesity prevention and reduction, is most effective in supporting optimal health, holistic wellness and academic achievement [23, 58–60].

Finally, the findings that the combined effects of lifestyle behaviors result in substantially higher likelihood of meeting expectations in all subjects is an important contribution to the literature. Ickovics et al. noted similar results in a sample of low-income American children of the same age – children with higher levels of ‘health assets’ including indicators of healthy diet, physical activity, screen time and sleep, were 2.2 times more likely to meet goals in mathematics, reading and writing compared to those who had the fewest health assets [25]. A Spanish study with the objective of investigating the combined effects of meeting recommendations for diet, physical activity, screen time and sleep on the self-reported grades of adolescents had similar findings for girls [17]. The present study complements and expands on these important findings drawing on a large, population-based sample of children. Collectively, these studies speak to the value of school-based health promotion initiatives that are more comprehensive in their approach compared to initiatives that address only singular aspects of health. Successful models of a comprehensive school health approach that have led to improvement in the healthfulness of children’s lifestyle behaviors have been well-evaluated [31, 61]. Interventions that aim to improve multiple lifestyle behaviors may have a greater impact on academic achievement than those that focus on single behaviors.

Of note is girls’ strong, positive association with the likelihood of meeting expectations for reading and writing. There was no gender effect for meeting expectations in mathematics. These results are consistent with other investigations about the school performance of girls and boys [62]. Stratified analyses by gender revealed no substantial differences between effects of individual lifestyle behaviors and academic achievement across gender (results not shown). Children’s test scores are influenced by a multitude of factors, and differential influences between girls and boys, including self-confidence and parental support for specific career streams, are among them [62]. Further investigation is merited to determine the cause of the substantial gender differences in academic achievement in this population.

This study has several key strengths. First, it is a very large, population-based sample of children who are representative of an entire Canadian province. This study evaluated lifestyle behaviors with respect to established recommendations, which allows for easier interpretation and specific targets for health promotion initiatives. Non-response weighting was employed in order to account for non-response of students residing in lower-income neighbourhoods in Nova Scotia. This study also used results from standardized exams from several different subjects, both eliminating bias from self-reported grades and illustrating that lifestyle behaviors may have differential effects for different cognitive tasks. This study used validated questionnaires to assess diet and physical activity as well as directly measured heights and weights. Limitations of the study were the self-reported nature of lifestyle behaviors that can be prone to bias. The time lag between measurement of lifestyle behaviors and the writing of standardized exams may have introduced error to estimates of association. In the case of screen time, only TV watching was captured in this analysis and other forms of screen-based media which may be widely used were not included. In addition, questions used to assess sleep and screen time where not validated. As the CLASS study aims to collect a wide breadth of information, shorter, non-validated questions were used on surveys to reduce participant burden. Though a validated questionnaire was used to assess physical activity levels, the PAQ-C does not provided information about duration or intensity of physical activity, which is thought to be important in terms of its influence on academic achievement and relationship with meeting recommendations. As such, a lack of association may be due to the limited information provided by the tool used. Provided the large scope of this study, objective measurements of lifestyle behaviors were not feasible. Finally, standardized tests are only one means by which to evaluate children’s academic success and their value has been disputed [63]. Investigations of the relationship between healthy lifestyle behaviors and other measures of academic success including enjoyment of school, psycho-social well-being, and sense of belonging are important complements to consider. There is also possibility of residual confounding by unmeasured variables including IQ and measures of mental health.

## Conclusions

This study demonstrates that individual lifestyle behaviors have independent, positive associations with academic achievement, and that cumulative effects of multiple healthy lifestyle behaviors have a stronger positive association with academic outcomes in Reading and Writing than any individual association. These findings suggest that school-based health promotion approaches that address multiple lifestyle behaviors instead of single behaviors may have more benefit for academic achievement. Future studies investigating a longitudinal link between lifestyle behaviors, body weight and academic achievement are important to strengthen the prospective findings of this study and others similar to it.

## Additional file

Additional file 1: Table S1. Correlations between Predictors of Interest: Meeting Recommendations for Lifestyle Behaviors among Grade 5 Students in Nova Scotia, Canada. (DOCX 69 kb)

## Abbreviations

BMI: Body mass index
CLASS: Children’s lifestyle and school-performance study
DQI: Diet quality index-international
IOTF: International obesity task force
PAQ-C: Physical activity questionnaire for children
YAQ: Harvard food frequency questionnaire for children and youth
