# Supplementary material for: The combined impact of diet, physical activity, sleep and screen time on academic achievement: a prospective study of elementary school students in Nova Scotia, Canada
Source: Int J Behav Nutr Phys Act. 2017 Mar 9;14:29. doi: 10.1186/s12966-017-0476-0 (PMC5343372; doi:10.1186/s12966-017-0476-0)
Supplement: Additional file 1: Table S1. — Correlations between Predictors of Interest: Meeting Recommendations for Lifestyle Behaviors among Grade 5 Students in Nova Scotia, Canada. (DOCX 69 kb) [file 12966_2017_476_MOESM1_ESM.docx]

| Variable | Vegetables and Fruits | Grain Products | Milk and Alt. | Meat and Alt. | Saturated Fat | Free Sugars | Physical Activity | Sleep | Screen  Time | Body Weight Status |
| --- | --- | --- | --- | --- | --- | --- | --- | --- | --- | --- |
| Vegetables and Fruits | 1.000 |  |  |  |  |  |  |  |  |  |
| Grain Products | -0.091 | 1.000 |  |  |  |  |  |  |  |  |
| Milk and Alternatives | -0.051 | -0.131 | 1.000 |  |  |  |  |  |  |  |
| Meat and Alternatives | -0.027 | -0.029 | -0.141 | 1.000 |  |  |  |  |  |  |
| Saturated Fat | 0.311 | 0.150 | -0.235 | -0.020 | 1.000 |  |  |  |  |  |
| Free Sugars | 0.186 | 0.164 | 0.180 | 0.120 | 0.006 | 1.000 |  |  |  |  |
| Physical Activity | 0.096 | -0.025 | 0.067 | 0.008 | 0.030 | 0.069 | 1.000 |  |  |  |
| Sleep | -0.008 | 0.002 | 0.009 | -0.025 | 0.021 | -0.015 | -0.006 | 1.000 |  |  |
| Screen Time | 0.051 | 0.009 | 0.062 | -0.011 | -0.007 | 0.102 | 0.043 | 0.239 | 1.000 |  |
| Body Weight Status | -0.025 | 0.017 | -0.004 | 0.021 | 0.024 | -0.042 | -0.081 | -0.018 | -0.085 | 1.000 |

**Supplementary Table 1: Correlations between Predictors of Interest : Meeting Recommendations for Lifestyle Behaviors among Grade 5 Students in Nova Scotia, Canada**
